# Association Between Non-obstructive Chronic Bronchitis and Incident Chronic Obstructive Pulmonary Disease and All-Cause Mortality: A Systematic Review and Meta-Analysis

**DOI:** 10.3389/fmed.2021.805192

**Published:** 2022-01-25

**Authors:** Fan Wu, Huanhuan Fan, Jing Liu, Haiqing Li, Weifeng Zeng, Silan Zheng, Heshen Tian, Zhishan Deng, Youlan Zheng, Ningning Zhao, Guoping Hu, Yumin Zhou, Pixin Ran

**Affiliations:** ^1^National Center for Respiratory Medicine, State Key Laboratory of Respiratory Disease, National Clinical Research Center for Respiratory Disease, Guangzhou Institute of Respiratory Health, The First Affiliated Hospital of Guangzhou Medical University, Guangzhou Laboratory, Guangzhou, China; ^2^The Third Clinical College, Department of Respiratory Medicine, The Third Affiliated Hospital of Guangzhou Medical University, Guangzhou, China; ^3^School of Public Health, Guangzhou Medical University, Guangzhou, China

**Keywords:** COPD, meta-analysis, non-obstructive chronic bronchitis, NOCB, systematic review

## Abstract

**Background:**

Chronic bronchitis in patients with chronic obstructive pulmonary disease (COPD) is associated with poor respiratory health outcomes. However, controversy exists around whether non-obstructive chronic bronchitis (NOCB) is associated with airflow obstruction, lung function decline, and all-cause mortality in ever smoker or never smoker.

**Research Question:**

This systematic review and meta-analysis aimed to clarify the relationship between NOCB and incident COPD, lung function decline, and all-cause mortality, and to quantify the magnitude of these associations.

**Study Design and Methods:**

We searched PubMed, Embase, and Web of Science for studies published up to October 1, 2021. Eligibility screening, data extraction, and quality assessment of the retrieved articles were conducted independently by two reviewers. Studies were included if they were original articles comparing incident COPD, lung function decline, and all-cause mortality in normal spirometry with and without chronic bronchitis. The primary outcomes were incident COPD and all-cause mortality. The secondary outcomes were respiratory disease-related mortality and lung function decline. Pooled effect sizes and 95% confidence intervals (CIs) were calculated using the random-effects model.

**Results:**

We identified 17,323 related references and included 14 articles. Compared with individuals without NOCB, individuals with NOCB had an increased risk of incident COPD (odds ratio: 1.98, 95% CI: 1.21–3.22, *I*^2^ = 76.3% and relative risk: 1.44, 95%CI: 1.13–1.85, *I*^2^ = 56.1%), all-cause mortality (hazard ratio [HR]: 1.38, 95%CI: 1.26–1.51, *I*^2^ = 29.4%), and respiratory disease-related mortality (HR: 1.88, 95%CI: 1.37–2.59, *I*^2^ = 0.0%). Data on the decline in lung function could not be quantitatively synthesized, but the five articles that assessed the rate of decline in lung function showed that lung function declines faster in individuals with NOCB. The mean difference in the additional decline in forced expiratory volume in 1 s ranged from 3.6 to 23.2 mL/year.

**Interpretation:**

Individuals with NOCB are at a higher risk of incident COPD and all-cause mortality than individuals without NOCB, highlighting the crucial need for strategies to screen for and reduce NOCB risk.

**Systematic Review Registration:**

https://www.crd.york.ac.uk/PROSPERO/ PROSPERO, identifier CRD42020202837

## Introduction

Chronic obstructive pulmonary disease (COPD) is a common, preventable, and treatable disease that is characterized by persistent respiratory symptoms and airflow obstruction. It is currently a leading cause of death and disability worldwide (1, 2). Identifying individuals who will eventually develop airflow obstruction that is consistent with a COPD diagnosis may enable therapeutic interventions with the potential to modify the disease course (3).

Chronic bronchitis is defined epidemiologically as cough and sputum production for ≥3 months each year for ≥2 consecutive years (4). The main risk factors for chronic bronchitis are similar to COPD, including smoking, secondhand smoke exposure, biofuel exposure, and occupational exposure (2, 5). The diagnoses of chronic bronchitis and COPD do not completely overlap. Chronic bronchitis not only occurs in patients with COPD but also in individuals with normal lung function, with prevalence estimates varying widely, both among individuals with normal lung function (2.2–17%) and among individuals with COPD (10.2–30%) in population-based studies (6–11). In 2001, the Global Strategy for the Diagnosis, Management, and Prevention of Chronic Obstructive Lung Disease (GOLD) report proposed an “at risk” stage (GOLD stage 0), which was defined by the presence of symptoms (chronic cough and sputum production) in the absence of spirometry abnormalities that cross the diagnostic threshold for COPD (12). This category was later abandoned in GOLD 2006 because not all of these individuals progressed to COPD (13). Moreover, controversy exists about whether non-obstructive chronic bronchitis (NOCB) is associated with incident airflow obstruction (10, 14).

Understanding the association between NOCB and incident COPD and respiratory health outcomes has important implications for disease management, such as in terms of targeted treatments and cautions about drug usage. Independent studies have shown a significantly increased risk of airflow obstruction and all-cause mortality in individuals with NOCB, and this has been summarized in a recent narrative review (3, 11, 15–17). However, to our knowledge, no studies have systematically synthesized this evidence. Therefore, we aimed to perform a systematic review and meta-analysis to evaluate NOCB and incident COPD and respiratory health outcomes to improve the statistical power, help to identify modest risk differences among study groups, and provide a solid basis to identify interventional strategies in the future.

## Methods

The study protocol was registered with the International Prospective Register of Systematic Reviews (registration number: CRD42020202837). This systematic review and meta-analysis was performed in accordance with Preferred Reporting Items for Systematic Reviews and Meta-Analyses (PRISMA) guidelines (18).

### Search Strategy and Selection Criteria

In this systematic review and meta-analysis, two reviewers (FH and JL) independently searched Embase, Web of Science, and PubMed to identify studies published from database inception to October 1, 2021. Keywords and subject terms were customized for each database. The search terms combined the respiratory symptoms related to chronic bronchitis (chronic cough, sputum production, phlegm, productive cough, and chronic mucus hypersecretion) and prognostic terms (airflow obstruction, COPD, mortality, and lung function). The references of related studies were also consulted to identify potentially relevant articles.

The eligibility of identified studies was independently verified, and disagreements were resolved by discussion or, where necessary, by consulting a third researcher (FW). For primary inspection, the titles and abstracts were reviewed, and articles were excluded mainly for COPD patients with chronic bronchitis and non-extractable data (qualitative, case report, or review articles). For secondary inspection, full-text review was performed, and articles were excluded based on the inclusion and exclusion criteria. Studies that satisfied the following criteria were included: (1) prospective cohort study or retrospective cohort study; (2) inclusion of odds ratios (ORs), relative risks (RRs), or hazard ratios (HRs) with corresponding 95% confidence intervals (CIs) to estimate COPD risk; the risk of mortality (all-cause mortality and respiratory disease-related mortality) in individuals with NOCB; or enough data to calculate these risks; (3) comparison of individuals without NOCB; (4) independent studies. Studies that were the same as the published dataset were not considered independent. We deemed studies as eligible if they were longitudinal cohort studies that enrolled adults and reported the association between NOCB and the study outcomes.

NOCB was defined as chronic cough and sputum production for ≥3 months for ≥2 consecutive years in subjects without airflow obstruction. Without NOCB was defined as normal spirometry and no chronic bronchitis. Without NOCB serves as a healthy control of NOCB, which does not limit smoking status. The primary study outcomes were incident COPD and all-cause mortality. The GOLD criterion (a post-bronchodilator forced expiratory volume in 1 s [FEV_1_]/forced vital capacity [FVC] ratio of <0.7) was the preferred definition of COPD (2). Other accepted definitions of COPD included a pre-bronchodilator FEV_1_/FVC ratio of <0.7 or an FEV_1_/FVC ratio less than the lower limit of normal (LLN). The secondary outcomes were respiratory disease-related mortality and lung function decline.

### Data Extraction and Quality Assessment

Two reviewers (FH and JL) independently extracted the study information and independently verified the quality of each study. The extracted information included first author, year of publication, location, study design, year of enrolment, study outcome, follow-up time, and the characteristics of the study participants (sample size, age, definition of chronic bronchitis, definition of normal spirometry). Two reviewers (HF and JL) independently conducted quality assessments of the included studies. The quality of each study was based on the Newcastle–Ottawa Scale (NOS) for cohort studies (19). Studies were considered to be good quality if the total score was at least 7 out of 9. Disagreements were resolved by discussion and further review.

### Data Synthesis and Analysis

The outcomes of the synthesis included the OR and the RR of incident COPD, the HR of all-cause mortality and respiratory disease-related mortality, and the mean difference in the rate of lung function decline. For eligible studies that reported the HR and 95% CI of incident COPD, the HR and 95% CI were pooled with the RR. We could use the calculation formula to convert the OR into the RR, but some studies did not report the proportion of the control group that developed COPD, and the OR of most studies was adjusted for confounding factors (20). Therefore, we decided to present both the OR and RR results. The random-effects model was used to calculate the pooled effect sizes and 95% CIs because the studies were conducted over a wide range of settings in different populations, such as the baseline characteristics of the participants, the follow-up duration, and adjustment for confounders (21). Specific subgroups (different smoking statuses and baseline ages) were examined. The *I*^2^ statistic was used to evaluate study heterogeneity. An *I*^2^ value of 0–24% was considered as no heterogeneity. Greater values represented greater heterogeneity, with values of 25–49% representing low heterogeneity, 50–74% representing moderate heterogeneity, and >75% indicating high heterogeneity (22). Publication bias was evaluated using funnel plots, Begg's tests, and Egger's tests (23). All statistical tests were two sided, and a *P-*value of < 0.05 was considered statistically significant. Stata/SE 15.1 (Statacorp LP, College Station, TX, USA) software was used for the meta-analysis.

## Results

**Figure 1:**
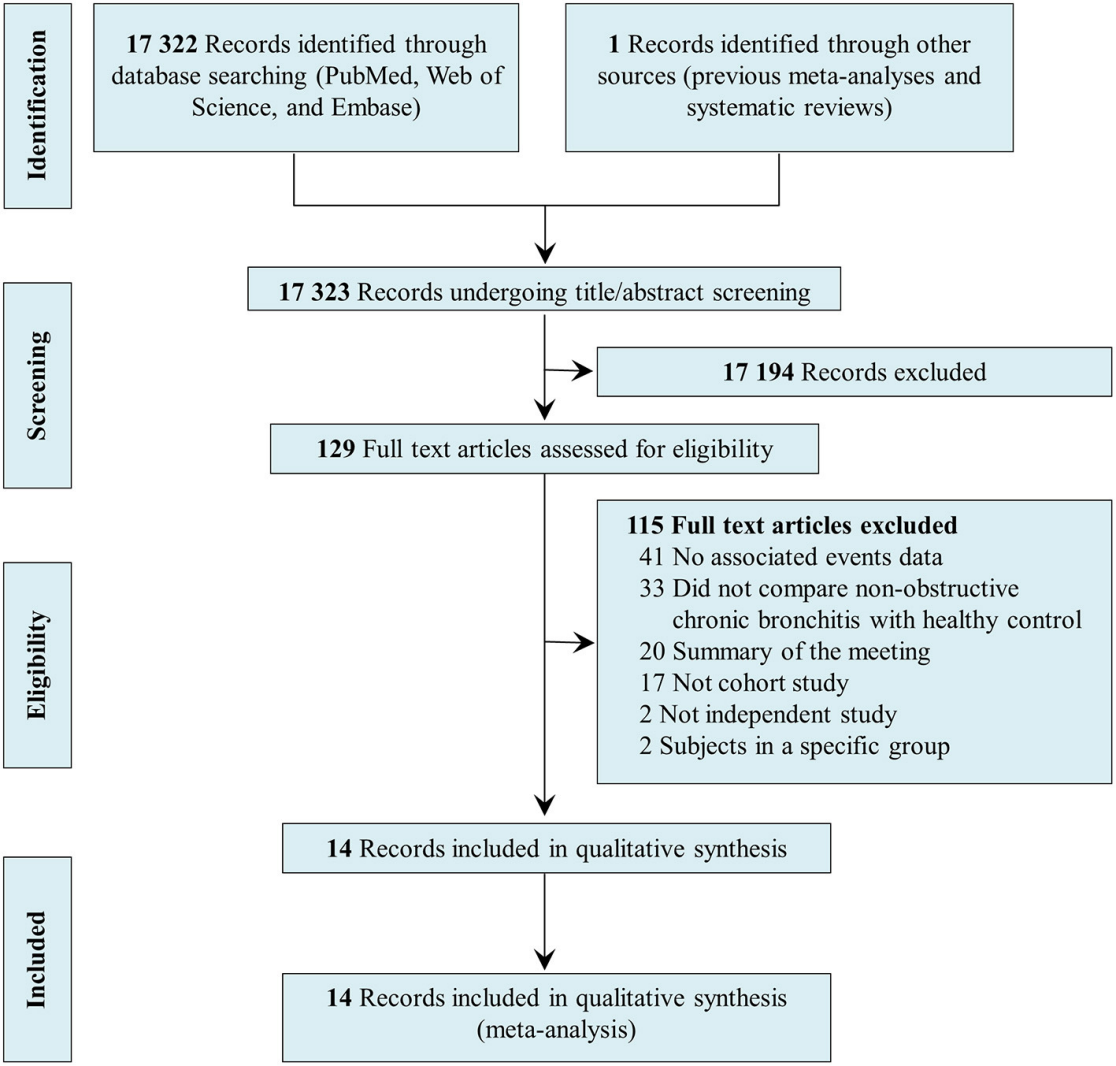
Preferred reporting items for systematic reviews and meta-analyses flow diagram of systematic search and selection.

Figure 1 shows the PRISMA flow diagram of the systematic search and selection, including the number of papers identified in PubMed, Embase, and Web of Science; the number of excluded studies; and the reason for study exclusion. Of the 17,323 records identified during the search, 129 records were selected for full-text review; 17,194 were excluded because the topic of this review was not evaluated. After reading the full texts, an additional 115 articles were excluded for the following reasons: no data on associated events, no comparison between individuals with NOCB and healthy control subjects, summary of the meeting, not a cohort study, not an independent study, subjects in a specific group. Finally, 14 studies were included in this systematic review and meta-analysis (10, 11, 14–16, 24–32).

### Study Characteristics and Quality Assessment

The key characteristics of the studies included in the meta-analysis are presented in Table 1. In total, 174,320 participants without COPD were included in 14 studies. The average follow-up time of eight studies was >10 years, the average follow-up time of four studies was between 5 and 10 years, and the average follow-up time of two studies was <5 years. Thirteen studies used a pre-bronchodilator FEV_1_/FVC ratio of ≥0.7 as the main definition of normal spirometry, and one study used a pre-bronchodilator FEV_1_/FVC ratio ≥ the LLN as the main definition of normal spirometry. Coronary Artery Risk Development in Young Adults Lung Study data were published in two articles (15, 30). In a study by Balte et al., the HR and 95% CI were used to calculate the risk of incident COPD (15). In a study by Kalhan et al., the OR and 95% CI were used to calculate the risk of COPD (30). The data from both articles were included in this study because we did not combine HR and OR data; rather, we presented the corresponding results separately.

**Table 1:** Characteristics of all studies included in the meta-analysis.

**References**	**Regions (Country)**	**Study design (cohort)**	**Chronic bronchitis** **definition**	**Normal spirometry definition**	**Sample size (% men)**	**Year of enrolment**	**Age (mean, years)**	**Follow-up (years)**
Vestbo et al. (10)	Copenhagen, Denmark	Prospective cohort	Productive cough (cough up phlegm for as much as 3 months every year)	Pre-bronchodilator FEV_1_/FVC≥0.7	11,207 (44%)	1976–1978	51.7	15
Mannino et al. (24)	America	Prospective cohort	Cough, sputum, wheeze	Pre-bronchodilator FEV_1_/FVC≥0.7	5,542 (45%)	1971–1975	47.6	17.9
Ekberg-Aronsson et al. (25)	Malmö, Sweden	Prospective cohort	Chronic bronchitis (cough and phlegm production on most days for >3 months in two or more consecutive years)	Pre-bronchodilator FEV_1_/FVC≥0.70 and FEV_1_≥80% predicted	22,044 (66.4%)	1974–1992	46.8	21.5
Lindberg et al. (31)	Norrbotten, Sweden	Prospective cohort	Chronic productive cough (have phlegm when coughing, or have phlegm which is difficult to bring up, most days for periods of at least 3 months, during at least the last 2 years)	Pre-bronchodilator FEV_1_/VC≥0.70	1,109	1986	NA	10
Stavem et al. (14)	Oslo, Norway	Prospective cohort	Productive cough (cough up phlegm for as much as 3 months every year)	Pre-bronchodilator FEV_1_/FVC≥0.7	1,619 (100%)	1972–1975	49.8 (5.5)	26
de Marco et al. (26)	12 countries in Europe	Prospective cohort	Chronic cough and phlegm	Pre-bronchodilator FEV_1_/FVC≥0.7	4,933 (48%)	1991–1993	20–44	8.9
Guerra et al. (27)	Tucson, America	Prospective cohort	Chronic bronchitis (cough and phlegm production on most days for >3 months in two or more consecutive years)	Pre-bronchodilator FEV_1_/FVC≥0.7	1,412 (42%)	1972–1973	49.1	22 years for incident COPD / 31 years for all-cause mortality
Yamane et al. (29)	Hiroshima, Japan	Retrospective-prospective cohort	Productive cough (cough and phlegm production on most days for >3 months in two or more consecutive years)	Pre-bronchodilator FEV_1_/FVC≥0.7 and VC≥80% predicted	783 (NA)	1993 and 2004	49.6	2.8
Probst-Hensch et al. (32)	Switzerland	Prospective cohort	cough or phlegm during the day or at night on most days for as much as 3 months each year for ≥2 years	Pre-bronchodilator FEV_1_/FVC≥0.7	765 (NA)	1991	NA	11
Brito-Mutunayagam et al. (28)	Adelaide, Australia	Retrospective cohort	Cough most or every day and/or sputum production	Post-bronchodilator FEV_1_/FVC≥0.7	3,547 (49.5%)	2000–2003	46.0 (0.3)	3.5
Allinson et al. (16)	England, Scotland, and Wales	Prospective cohort	Chronic mucus hypersecretion (chronic cough with chronic sputum expectoration at least 3 months yearly)	Pre-bronchodilator FEV_1_/FVC≥lower limit of normal	1,079	1989	43	17–21
Kalhan et al. (30)	Birmingham, Chicago, Minneapolis, Oakland, America.	Prospective cohort	had periods or episodes of (increased) cough and phlegm lasting for 3 weeks or more each year	Pre-bronchodilator FEV_1_/FVC≥0.7	2,749 (42.8%)	1985–2016	25.1	30
Colak et al. (11)	Copenhagen, Denmark	Prospective cohort	Chronic mucus hypersecretion (cough up phlegm as long as three consecutive months each year)	Pre-bronchodilator FEV_1_/FVC≥0.7	97,955 (45%)	2003–2015	57	8.8
Balte et al. (15)	America	Prospective cohort	Chronic bronchitis (cough and phlegm for at least 3 months for 2 or more consecutive years)	Pre-bronchodilator FEV_1_/FVC≥0.7	22 325 (41.8%)	1971–2007	53.0 (16.3)	9.8

*FEV_1_, forced expiratory volume in 1 second; FVC, forced vital capacity; NA, not available*.

All studies were published between 2002 and 2020. The methodological quality of the included studies was satisfactory, with NOS nine-point quality assessment scores of between 6 and 9. One study was graded as being of fair quality, while all other studies were graded as being of good quality. The details of the quality assessment are presented in Table 2.

**Table 2:** Newcastle–Ottawa Scale scores and quality assessment of all studies included in the meta-analysis.

**References**	**Selection (stars awarded)**				**Comparability (stars awarded)**	**Outcome (stars awarded)**			**Quality** **(total stars** **awarded)**
	**Representativeness**	**Selection**	**Ascertainment**	**Outcome**		**Assessment**	**Follow-up**	**Adequacy**	
Vestbo et al. (10)	*	*	*	*	**	*	*	*	Good (9)
Mannino et al. (24)		*	*	*	**	*	*	*	Good (8)
Ekberg-Aronsson et al. (25)	*	*	*	*	*	*	*	*	Good (8)
Lindberg et al. (31)	*	*	*	*	**	*	*	*	Good (9)
Stavem et al. (14)	*	*		*	**	*	*	*	Good (8)
de Marco et al. (26)	*	*	*	*	**	*	*	*	Good (9)
Guerra et al. (27)	*	*	*	*	*	*	*	*	Good (8)
Yamane et al. (29)		*	*	*		*	*	*	Fair (6)
Probst-Hensch et al. (32)	*	*	*	*	*	*	*	*	Good (9)
Brito-Mutunayagam et al. (28)	*	*	*	*	**	*	*	*	Good (9)
Allinson et al. (16)	*	*	*	*	*	*	*	*	Good (8)
Kalhan et al. (30)	*	*	*	*	**	*	*	*	Good (9)
Colak et al. (11)	*	*	*	*	**	*	*	*	Good (9)
Balte et al. (15)	*	*	*	*	**	*	*	*	Good (9)

### Association Between NOCB and Incident COPD

**Figure 2:**
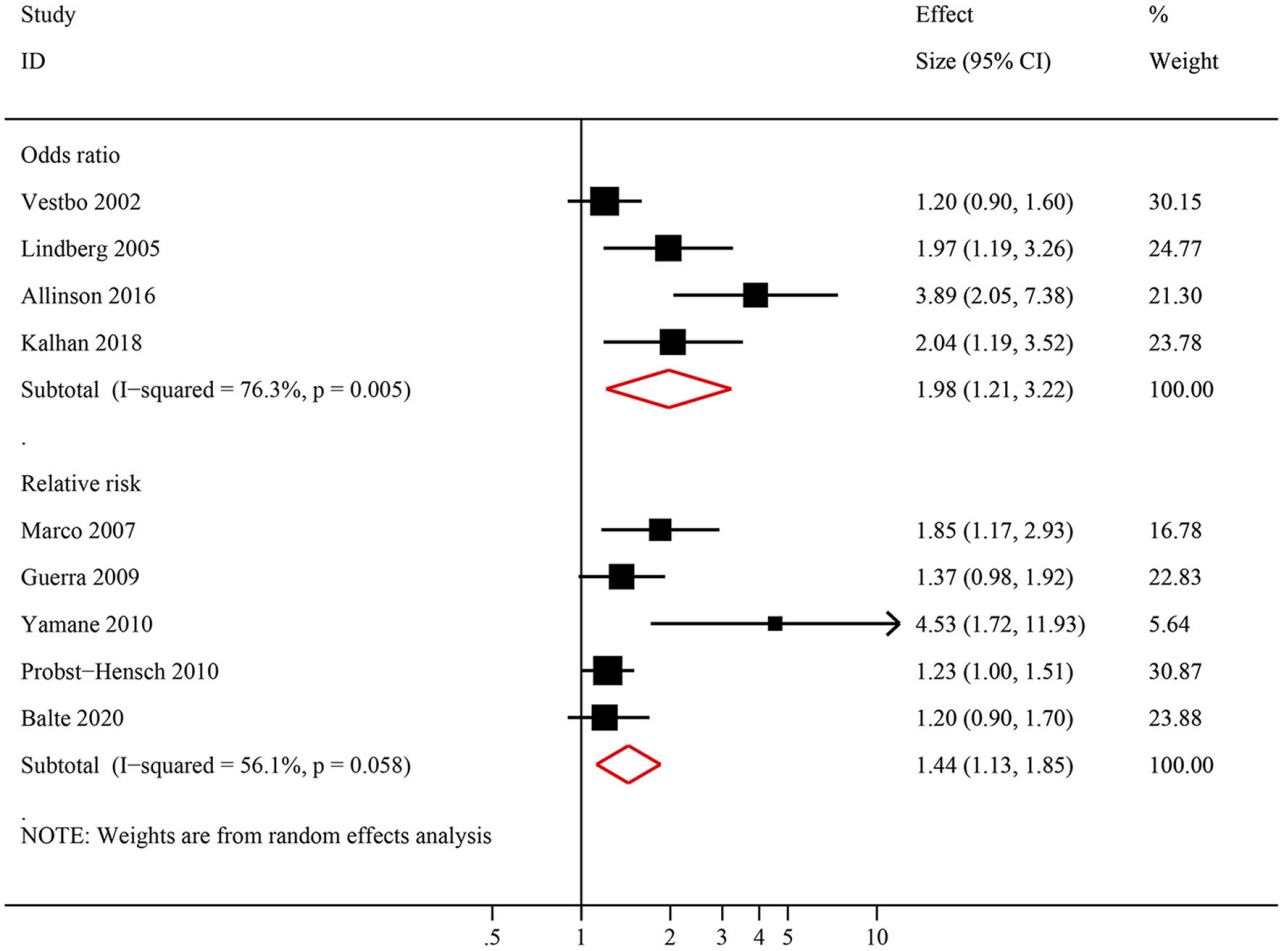
Forest plot of the risk of incident chronic obstructive pulmonary disease in individuals with non-obstructive chronic bronchitis compared with individuals without non-obstructive chronic bronchitis. Without non-obstructive chronic bronchitis was defined as normal spirometry and no chronic bronchitis.

Nine studies examined the association between NOCB and incident COPD. Among them, four studies presented the results as ORs and corresponding 95% CIs, two studies presented the results as RRs and corresponding 95% CIs, and three studies presented the results as HRs and corresponding 95% CIs. For the three studies that reported the HRs and 95% CIs of incident COPD, the HRs and 95% CIs were pooled with the RRs. Compared with normal spirometry without chronic bronchitis, the pooled analysis identified a significant increase in the risk of incident COPD in individuals with NOCB (OR: 1.98, 95% CI: 1.21–3.22, *P* = 0.006 and RR: 1.44, 95% CI: 1.13–1.85, *P* = 0.004) with significant inter-study heterogeneity (*I*^2^ = 76.3%, tau^2^ = 0.184, *P* = 0.005 and *I*^2^ = 56.1%, tau^2^ = 0.041, *P* = 0.058, respectively) (Figure 2). The number of studies included in this meta-analysis did not meet the minimum requirements for evaluating publication bias (10 articles). Therefore, although the publication bias evaluation was set in advance, it was not carried out.

### Association Between NOCB and All-Cause Mortality/Respiratory Disease-Related Mortality

**Figure 3:**
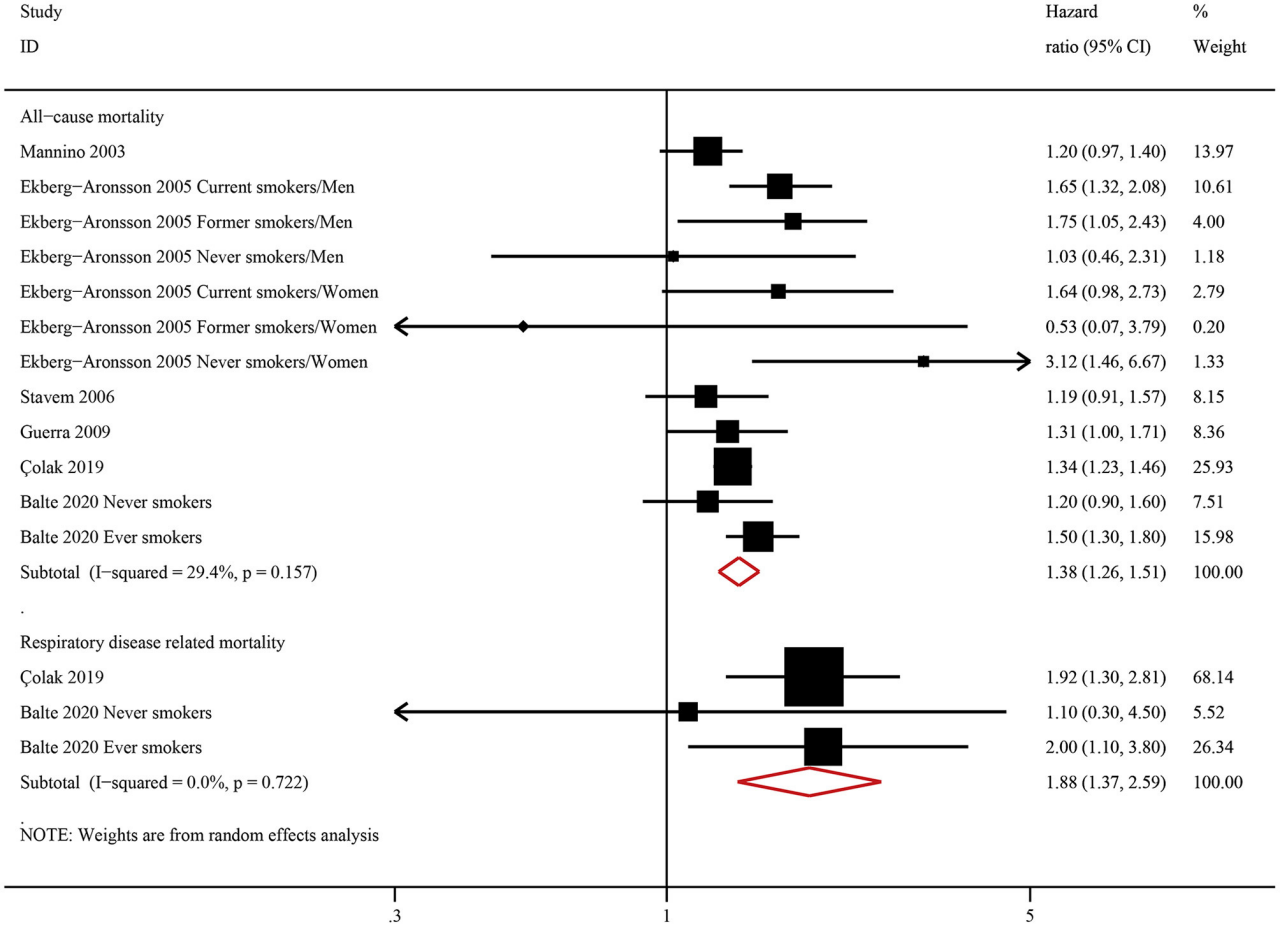
Forest plot of the risk of all-cause mortality and respiratory-related mortality in individuals with non-obstructive chronic bronchitis compared with individuals without non-obstructive chronic bronchitis. Without non-obstructive chronic bronchitis was defined as normal spirometry and no chronic bronchitis.

Six studies examined the association between NOCB and all-cause mortality. Compared with normal spirometry without chronic bronchitis, NOCB was associated with an increased risk of all-cause mortality (HR: 1.38, 95% CI: 1.26–1.51, *P* < 0.001) with low inter-study heterogeneity (*I*^2^ = 29.4%, Tau^2^ = 0.006, *P* = 0.157) (Figure 3). Two studies examined the association between NOCB and respiratory disease-related mortality. Compared with normal spirometry without chronic bronchitis, NOCB was associated with an increased risk of respiratory disease-related mortality (HR: 1.88, 95% CI: 1.37–2.59, *P* < 0.001) with no inter-study heterogeneity (*I*^2^ = 0.0%, Tau^2^ < 0.001, *P* = 0.722) (Figure 3).

### Narrative Summary of Selected Studies and Lung Function Decline

Data on the decline in lung function was not suitable for quantitative synthesis, but the five articles that assessed the rate of decline in lung function all suggested that lung function declines faster in individuals with NOCB than in those without. In the Copenhagen City Heart Study, FEV_1_ declined significantly faster in individuals with NOCB than normal spirometry in men without chronic bronchitis, but there was no significant difference in women (10). In a study of Japanese men, the absolute values of FEV_1_ and FEV_1_/FVC, and FEV_1_% predicted reduction in subjects with NOCB were significantly higher than those in subjects without NOCB (50.64 vs. 27.46 mL/year, 0.74 vs. 0.03%/year, and 0.61 vs. 0.39%/year, respectively) (29). In the North West Adelaide Health Cohort Study, the annual decrease in FEV_1_ in subjects classified as persistent GOLD stage 0 was significantly higher than that of people who did not experience symptoms of cough and sputum production (28). In the National Survey of Health and Development Study, chronic mucus hypersecretion was associated with both a lower FEV_1_ and a faster decline in FEV_1_ (16). The most recent selected publication (the National Heart, Lung, and Blood Institute Pooled Cohorts Study) found that participants with NOCB had an accelerated decrease in FEV_1_ (4.1 mL/year; 95% CI: 2.1–6.1 mL/year) and FVC (4.7 mL/year; 95% CI: 2.2–7.2 mL/year) among ever smokers (15).

### Subgroup Analyses

Table 3 shows the predefined subgroup analysis of incident COPD. The number of studies in each subgroup was too small to obtain clear research results. In short, NOCB in participants aged <50 years was significantly related to incident COPD.

**Table 3:** Association between non-obstructive chronic bronchitis and incident chronic obstructive pulmonary disease in subgroups.

**Subgroup**	**Number of studies**	**Effect** **size type**	**Effect** **size**	**95% CI**	***P*** **-** **value**	**Heterogeneity test**
<50 years old	2	OR	2.75	1.46–5.17	0.002	*I*^2^ = 56.0%, Tau^2^ = 0.117, *P* = 0.132
	2	RR	2.01	1.43–2.84	<0.001	*I*^2^ = 0.0%, Tau^2^ <0.001, *P* = 0.589
≥50 years old	1	OR	1.14	0.77–1.72	-	-
	1	RR	0.92	0.59–1.43	-	-
Never smokers	1	OR	5.14	1.28–20.59	-	-
	1	RR	0.86	0.27–2.72	-	-
Ever smokers	2	OR	1.89	0.67–3.55	0.227	*I*^2^ = 77.7%, Tau^2^ = 0.581, *P* = 0.011
	2	RR	4.10	0.74–22.69	0.106	*I*^2^ = 89.4%, Tau^2^ = 2.003, *P* <0.001

*COPD, chronic obstructive pulmonary disease; CI, confidence interval; OR, odds ratio; RR, relative risk*.

**Figure 4:**
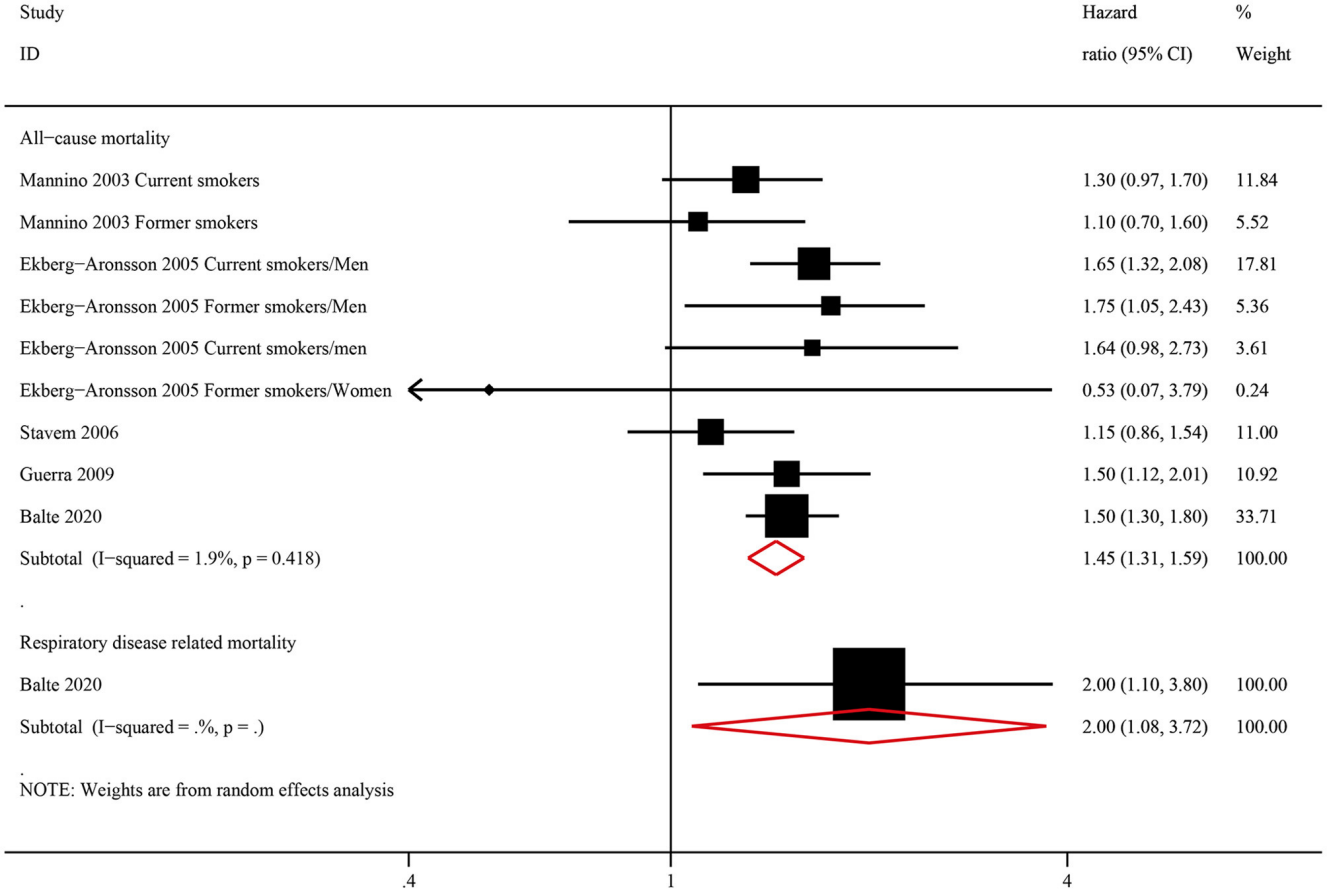
Forest plot of the risk of all-cause mortality and respiratory-related mortality in ever smokers with non-obstructive chronic bronchitis compared with ever smokers without non-obstructive chronic bronchitis. Without non-obstructive chronic bronchitis was defined as normal spirometry and no chronic bronchitis.

**Figure 5:**
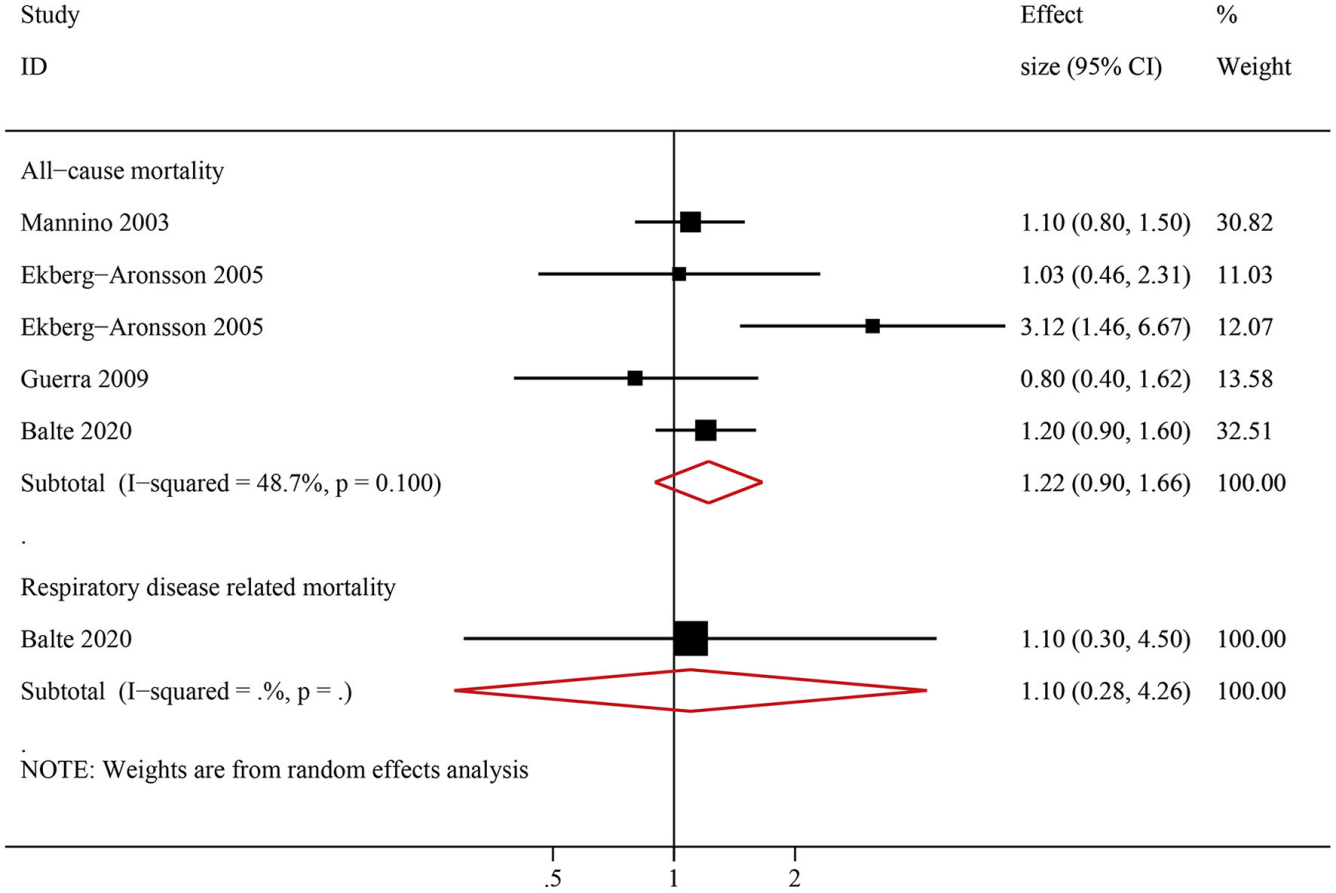
Forest plot of the risk of all-cause mortality and respiratory-related mortality in never smokers with non-obstructive chronic bronchitis compared with never smokers without non-obstructive chronic bronchitis. Without non-obstructive chronic bronchitis was defined as normal spirometry and no chronic bronchitis.

Among ever smokers, NOCB was associated with a higher all-cause mortality (HR: 1.45, 95% CI: 1.31–1.59, *P* < 0.001) and respiratory disease-related mortality (HR: 2.00, 95% CI: 1.08–3.72, *P* < 0.001) than in individuals with normal lung spirometry without NOCB (Figure 4). Furthermore, among never smokers, individuals with NOCB did not demonstrate a significant difference in all-cause mortality (HR: 1.22, 95% CI: 0.90–1.66, *P* = 0.206) or respiratory disease-related mortality (HR: 1.10, 95% CI: 0.28–4.26, *P* = 0.890) compared with individuals with normal spirometry and without NOCB (Figure 5).

## Discussion

To our knowledge, this systematic review and meta-analysis is the first to quantitatively synthesize the current evidence on NOCB and respiratory health outcomes, further expanding our knowledge in this area. The main finding from our systematic review and meta-analysis is that individuals with NOCB experience a moderately elevated risk of incident COPD, all-cause mortality, and respiratory disease-related mortality, as well as faster lung function decline.

According to the current definition of chronic bronchitis, the prevalence of chronic bronchitis in the population is relatively high, and the burden of disease is also high (7). Our study demonstrated that NOCB is associated with incident COPD and all-cause mortality. These results emphasize the importance of recognizing NOCB as a pre-COPD phenotype and as an indication for lung function testing to screen for COPD (3). Accordingly, we should reconsider the strategy of early diagnosis and prevention of COPD by paying more attention to respiratory symptoms. It is promising that a double-blinded placebo-controlled follow-up clinical trial of inhalational indacaterol/glycopyrrolate (27.5/15.6 mcg twice daily) for the treatment of subjects with a COPD Assessment Test score of >10 and an FEV_1_/FVC ratio of >0.7 is currently underway (33).

Not all individuals with NOCB will progress to COPD; rather, the classification identifies an especially at-risk population that should be more closely followed up for risk management. Small airway dysfunction (34, 35), a reduced diffusion capacity (36), an accelerated decline in FEV_1_ (37), and emphysema (38) are also indicators of an increased risk of incident COPD. A multi-directional comprehensive assessment to identify groups that are high-risk for COPD should be performed (3, 39). We should also note that many participants with NOCB may not necessarily progress to COPD; instead, chronic cough and sputum production are also clinical symptoms of other diseases, such as bronchiectasis, pulmonary tuberculosis, and coronavirus disease 2019, amongst others. For individuals with NOCB, we should also screen for other organic diseases so as not to affect the diagnosis and treatment of these diseases.

Our predefined subgroup analysis found that NOCB in participants aged <50 years was associated with a higher risk of incident COPD. Two studies involving participants aged >50 years did not show that NOCB is associated with incident COPD. Due to the small number of related studies, no clear conclusion can yet be reached. However, this knowledge should inspire future research, and age stratification is needed. Further research is needed to enhance our understanding of the natural history of NOCB, while further supporting preventative and therapeutic approaches (16). Furthermore, our predefined subgroup analysis demonstrated that NOCB is associated with all-cause mortality and respiratory disease-related mortality in ever smokers, but NOCB is not associated with all-cause mortality and respiratory disease-related mortality in never smokers. This result underscores the importance of evidence-based risk factor optimization in patients with NOCB, including smoking avoidance and cessation (15).

The symptoms of cough and sputum production associated with chronic bronchitis fluctuate, and they are affected by certain factors, such as smoking. De Marco et al. analyzed European Community Respiratory Health Survey data and found that in 62.0% of subjects with chronic cough/phlegm production, symptoms were relieved in the 10-year follow-up (26). Relief of chronic cough and sputum production may be associated with a better prognosis than persistent chronic cough and sputum production. However, individuals with these symptoms are still more likely to progress to COPD than subjects who continue to be asymptomatic (26). Therefore, although symptoms are relieved in some individuals, they should not be disregarded; instead, follow-up and COPD screening should be strengthened.

Although our study was not designed to confirm the pathophysiologic link between NOCB and incident COPD, and we cannot thus assign directional causality, several plausible mechanisms can be proposed. Chronic sputum production, as one of the symptoms of chronic bronchitis, is caused by increased mucus secretion. The most intuitive effect of excessive mucus secretion in the airway is the formation of mucus plugs that block the airway, resulting in decreased lung function. Previous research has shown that computed tomography-identified mucus plugging is associated with mortality in smokers with and without COPD (40). Moreover, an increase in the concentration of MUC5AC is negatively correlated with FEV_1_ (41). MUC5AC-containing regions are tethered to the epithelium, which causes impaired mucociliary transport (42). The unclearable intrapulmonary mucus plugs contribute to prospective exacerbation (43). With exacerbation attacks, lung function demonstrates a further decline (44). In addition, the mechanism of increased mucus secretion is also related to the pathogenesis of COPD. The activation of non-type-2 pathways (interleukin (IL)-1β and IL-17A) upregulates the production of MUC5AC and MUC5B (45). These two inflammatory factors have also demonstrated involvement in the occurrence of COPD (46, 47).

There are some potential limitations in this study that should be noted. First, heterogeneity was high in our meta-analysis of incident COPD. Therefore, the quantitative analysis results of incident COPD in this study should be interpreted carefully. Second, our study was originally set to conduct a subgroup analysis of different smoking statuses and different baseline ages to reduce heterogeneity, but there were too few relevant studies to perform a predefined subgroup analysis. There is still an urgent need for further large-sample cohort studies to evaluate the association between NOCB and incident COPD and respiratory health outcomes in specific subgroups (ever smokers, never smokers, patients aged <50 years, patients aged ≥50 years, and patients with fluctuating symptoms of chronic bronchitis). Finally, almost all studies included in our meta-analysis used pre-bronchodilator lung function data to diagnose COPD. A previous study has demonstrated that pre-bronchodilator lung function overestimates the prevalence of COPD compared with post-bronchodilator lung function (48). This may have affected the estimation of incident COPD risk. Nevertheless, pre-bronchodilator measures are highly correlated with post-bronchodilator measures in the general population (49, 50). Despite this, future studies using post-bronchodilator measures to diagnose COPD are required.

## Interpretation

To conclude, this systematic review and meta-analysis demonstrated that NOCB is associated with an increased risk of incident COPD, all-cause mortality, and respiratory disease-related mortality, as well as faster lung function decline. Individuals with NOCB should be identified early through screening, and strategies aimed at controlling NOCB should be implemented.

## Data Availability Statement

The original contributions presented in the study are included in the article/supplementary material, further inquiries can be directed to the corresponding authors.

## Author Contributions

PR, YZ, FW, HF, and JL contributed to study conception, protocol of the review, and interpretation of data. FW, HF, JL, SZ, and WZ performed the systematic review. FW and HF performed the statistical analysis. YZ, PR, FW, HF, and JL drafted the paper and all authors provided critical revisions and contributed to the editing of the paper. All authors are guarantors of this work.

## Funding

This study was supported by the National Key Research and Development Program (2016YFC1304101), the Local Innovative and Research Teams Project of Guangdong Pearl River Talents Program (2017BT01S155), the National Natural Science Foundation of China (81970045), and Zhongnanshan Medical Foundation of Guangdong Province (ZNSA-2020003, ZNSA-2020012, and ZNSA-2020013).

## Conflict of Interest

The authors declare that the research was conducted in the absence of any commercial or financial relationships that could be construed as a potential conflict of interest.

## Publisher's Note

All claims expressed in this article are solely those of the authors and do not necessarily represent those of their affiliated organizations, or those of the publisher, the editors and the reviewers. Any product that may be evaluated in this article, or claim that may be made by its manufacturer, is not guaranteed or endorsed by the publisher.

## Abbreviations

COPD: chronic obstructive pulmonary disease
CI: confidence interval
FEV_1_: forced expiratory volume in 1 s
FVC: forced vital capacity
GOLD, Global Strategy for the Diagnosis, Management: and Prevention of Chronic Obstructive Lung Disease
HR: hazard ratio
LLN: lower limit of normal
NOCB: non-obstructive chronic bronchitis
NOS: Newcastle-Ottawa quality assessment scale
OR: odds ratio
PRISMA: Preferred Reporting Items for Systematic Reviews and Meta-Analyses
RR: relative risk.
